# Investigating the efficacy of integrated cognitive behavioral therapy for adult treatment seeking substance use disorder patients with comorbid ADHD: study protocol of a randomized controlled trial

**DOI:** 10.1186/1471-244X-13-132

**Published:** 2013-05-10

**Authors:** Katelijne van Emmerik–van Oortmerssen, Ellen Vedel, Maarten W Koeter, Kim de Bruijn, Jack J M Dekker, Wim van den Brink, Robert A Schoevers

**Affiliations:** 1 Arkin Mental Health Care and Addiction Treatment Center, Amsterdam, The Netherlands; 2 Jellinek Substance Abuse Treatment Center, Amsterdam, The Netherlands; 3 Amsterdam Institute for Addiction Research, Department of Psychiatry, Academic Medical Centre, University of Amsterdam, Amsterdam, The Netherlands; 4 Department of Psychiatry, University Medical Center Groningen, University of Groningen, Groningen, The Netherlands

**Keywords:** ADHD, SUD, Cognitive behavioral therapy, Adult, Integrated treatment

## Abstract

**Background:**

Attention deficit hyperactivity disorder (ADHD) frequently co-occurs with substance use disorders (SUD). The combination of ADHD and SUD is associated with a negative prognosis of both SUD and ADHD. Pharmacological treatments of comorbid ADHD in adult patients with SUD have not been very successful. Recent studies show positive effects of cognitive behavioral therapy (CBT) in ADHD patients without SUD, but CBT has not been studied in ADHD patients with comorbid SUD.

**Methods/design:**

This paper presents the protocol of a randomized controlled trial to test the efficacy of an integrated CBT protocol aimed at reducing SUD as well as ADHD symptoms in SUD patients with a comorbid diagnosis of ADHD. The experimental group receives 15 CBT sessions directed at symptom reduction of SUD as well as ADHD. The control group receives treatment as usual, i.e. 10 CBT sessions directed at symptom reduction of SUD only. The primary outcome is the level of self-reported ADHD symptoms. Secondary outcomes include measures of substance use, depression and anxiety, quality of life, health care consumption and neuropsychological functions.

**Discussion:**

This is the first randomized controlled trial to test the efficacy of an integrated CBT protocol for adult SUD patients with a comorbid diagnosis of ADHD. The rationale for the trial, the design, and the strengths and limitations of the study are discussed.

**Trial registration:**

This trial is registered in http://www.clinicaltrials.gov as NCT01431235.

## Background

Adult Attention Deficit Hyperactivity Disorder (ADHD) is highly frequent in Substance Use Disorder (SUD) patients [1,2]. SUD patients with comorbid ADHD start abusing substances at a younger age, use more substances and are hospitalized more often than SUD patients without ADHD [3]. ADHD is also associated with higher relapse rates after a SUD treatment for cocaine dependence [4] and alcohol dependence [5]. This results in suboptimal outcomes of SUD treatment in this population. At the same time, treatment of ADHD is compromised in the presence of SUD. Most ADHD treatment studies using methylphenidate in SUD patients have shown that this medication was not effective in reduction of ADHD symptoms [6-11], and only one randomized controlled trial reported some decrease in self-reported ADHD symptoms after methylphenidate treatment of ADHD in SUD patients [12]. None of the studies showed a clear effect on substance use outcomes. Similarly, atomoxetine was not superior to placebo in an RCT among adolescents with ADHD and SUD (mainly cannabis, alcohol and/or nicotine dependence) [13]. However, Wilens and colleagues found a significant decrease of ADHD symptoms when they compared atomoxetine with placebo in adult alcohol dependent patients with ADHD [14]. Again, there was no significant effect on alcohol use.

Other treatment options for ADHD such as cognitive behavioral therapy (CBT) or EEG neurofeedback have not been investigated yet in ADHD patients with a comorbid SUD. However, three recent randomized controlled trials reported a positive effect of CBT in adult ADHD patients without substance abuse [15-17]. In the study by Safren et al. [15], 86 adult ADHD patients with residual ADHD symptoms during medication treatment were randomized to individual CBT or to relaxation as a control condition. Assessments of ADHD symptoms by blinded investigators took place at baseline, post-treatment, and at 6 months and 12 months follow up. CBT resulted in a significant greater reduction of ADHD symptoms than relaxation therapy, both post-treatment and at 1 year follow up. In another study, Solanto et al. [16] investigated a meta-cognitive group therapy designed to improve time management, organization and planning in adults with ADHD. A total of 88 patients were stratified by medication use and randomized to the meta-cognitive therapy or a supportive psychotherapy group. Meta-cognitive therapy yielded significantly greater improvements in ADHD symptoms (self-rated, observer-rated by partner or family member, or rated by a blind evaluator) than supportive therapy. Finally, in the study by Emilsson et al. [17], 54 adult ADHD patients who were already on medication were randomized to a CBT based group program or to treatment as usual. Medium to large treatment effect sizes were found for evaluator-rated and self-rated ADHD symptoms at the end of treatment, which increased further at three months follow up. In addition, comorbid problems such as depression and anxiety symptoms improved at follow-up with large effect sizes.

The current study is designed to test the efficacy of an integrated CBT protocol combining a standardized motivational interviewing and coping skills training program for SUD with a CBT program for ADHD. The CBT program for SUD is based on evidence-based CBT protocols addressing substance abuse [18,19] adapted for use in the Netherlands [20,21], whereas the CBT program for ADHD is a series of adapted sessions from the treatment manual by Safren et al. [22,23]. This latter treatment manual was chosen because it was the only available evidence-based individual CBT protocol for ADHD at that moment.

Apart from ADHD outcomes, we are also interested in the potential effects of this integrated treatment on substance use. According to the self-medication hypothesis [24], substances are (also) used to alleviate distress caused by psychiatric disorders; this implies that a reduction of symptoms of ADHD could lead to an additional reduction in substance use compared to regular CBT for SUD. Since impulsivity is related to drug use [25], ADHD treatment could also result in reduced substance use because of a decline of impulsivity symptoms. Finally, effects on anxiety and depressive symptoms, quality of life and cost-effectiveness of the integrated treatment protocol are examined.

### Aims of the trial

The aims of this trial are to test the acceptance, feasibility, efficacy and cost-effectiveness of an individual integrated CBT protocol for SUD patients with a comorbid diagnosis of adult ADHD. The integrated CBT protocol aims to address both SUD and ADHD.

The primary research question is:

1. Does adding a CBT program aimed at reducing ADHD symptoms to a cognitive behavioral treatment as usual for SUD (TAU), result in a decrease of self-reported ADHD symptoms in adults with SUD and comorbid adult ADHD compared to TAU only at the end of treatment and at two months follow-up?

Secondary research questions are:

1. Does adding a CBT program aimed at reducing ADHD symptoms to TAU result in a greater reduction of self-reported substance use in adults with SUD and comorbid adult ADHD than TAU only?

2. Does adding a CBT program aimed at reducing ADHD symptoms to TAU result in a greater decrease of self-reported depression and anxiety and a greater increase in quality of life than TAU only?

3. Does adding a CBT program aimed at reducing ADHD symptoms to TAU result in a greater improvement in neuropsychological functions than TAU only?

4. What are the comparative costs per gained quality adjusted life year (QALY) for the integrated CBT protocol and TAU only?

5. Are baseline characteristics (e.g. performance on neuropsychological tasks) predictive of treatment response to either TAU or integrated CBT (patient-treatment matching)?

We hypothesize that patients in the integrated treatment condition will achieve stronger reductions in ADHD symptoms than patients in the TAU only condition at the end of treatment and at 2 months follow-up. Moreover, we expect participants in the integrated treatment condition to have lower scores on self-reported substance use, depression and anxiety and higher scores on quality of life than participants in the TAU only condition at the same time points. We also expect the integrated CBT protocol to result in greater improvements in performance on neuropsychological tasks, and we expect the integrated CBT protocol to have a higher cost-utility than TAU only. At this moment, we have no explicit hypothesis about the baseline characteristics that might be predictive of treatment response in terms of a decrease of ADHD symptoms in the integrated treatment condition or the TAU condition (patient-treatment matching).

## Methods

### Participants

#### Inclusion criteria

Participants are (self)referrals seeking treatment for their substance use problems at the Jellinek, a large addiction treatment center in Amsterdam, the Netherlands. To be eligible for the study, patients have to meet the following inclusion criteria: after intake allocated to outpatient treatment unit, aged 18–65 years, full command over the Dutch language, current DSM-IV diagnosis of any substance use disorder other than nicotine dependence only, and a comorbid DSM-IV diagnosis of ADHD with persisting symptoms meeting diagnostic criteria in adulthood. Patients with pathological gambling and other behavioral addictions are not included.

#### Exclusion criteria

Patients with severe neurological (e.g. dementia, Parkinson’s disease) or psychiatric disorders (e.g. psychosis, bipolar disorder) requiring medication, are excluded from the study. Patients with a borderline personality disorder are also excluded and referred to adequate treatment for this condition. Patients currently using ADHD medication (e.g. methylphenidate) are allowed to participate provided that they are on a stable dose and no medication changes are planned for the duration of the trial.

### Design and procedure

#### Recruitment and consent

During the standardized intake and treatment allocation procedure at the Jellinek, patients are screened for ADHD. Screen positive patients are invited for a semi-structured diagnostic interview with a specially trained psychologist to assess the presence of a DSM-IV diagnosis of adult ADHD. If ADHD, persisting in adulthood, is diagnosed, the patient is informed about the possible treatment options and receives oral and written information on the treatment study. If the patient is interested in participation, he or she is contacted by telephone by one of the investigators for further information. If the patient wants to participate in the study, informed consent is signed during the next visit. In the current study, no (additional) ADHD medication is provided.

#### Randomization and treatment allocation

Patients are randomized to receive either Treatment As Usual directed at the treatment of SUD (TAU only) or TAU plus CBT sessions aimed at reducing ADHD symptoms (integrated treatment condition). Treatment allocation is performed randomly by online application of a biased-coin randomization (minimization). In this way, we aim to ensure that trial arms are balanced with respect to three baseline characteristics: gender, use of ADHD medication (yes/no), and type of SUD diagnosis (alcohol only versus drugs). Neither patients nor therapists or investigators are blinded for the treatment allocation.

#### Procedure

**Figure 1:**
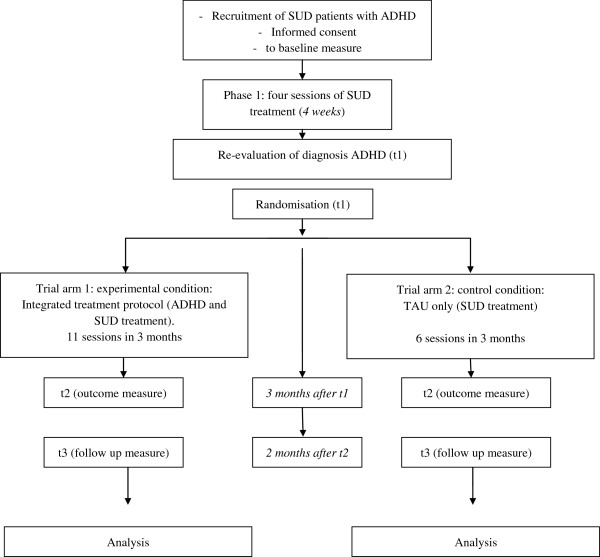
Trial flowchart.

Figure 1 provides an overview of the trial flow. Diagnostic assessment of SUD (CIDI), and screening and diagnostic assessment of ADHD (ASRS and CAADID; description of all three measures see below) take place at t-1. After informed consent and baseline assessment (t0), all participating patients start with phase 1 of the SUD treatment (four weekly sessions). During this treatment phase patients are motivated and stimulated to reach full abstinence in order to validate the ADHD diagnosis, i.e. a diagnosis not distorted by the presence of intoxication or withdrawal symptoms. The second ADHD assessment (CAADID), after the fourth session, is performed by another investigator. If the original ADHD diagnosis is confirmed, randomization takes place (t1). Following randomization, patients in the TAU only condition receive another six standard SUD treatment sessions in the course of the next three months (resulting in a total offer of 10 CBT sessions directed at treatment of SUD), whereas patients in the integrated treatment condition receive another 11 treatment sessions on both SUD treatment and ADHD treatment in the next three months (resulting in a total offer of 15 CBT sessions directed at treatment of both SUD and ADHD). At the end of treatment, all participants are assessed again (t2). A follow up assessment (t3) is performed two months after the last treatment session. Finally, participants in the TAU only condition are offered five ADHD treatment sessions after the follow up assessment (two months after end of treatment) as a compassionate treatment offer.

### Treatment protocols

Participants in the TAU only condition receive outpatient substance abuse treatment using a treatment program for SUD that is implemented nationally in the Netherlands [20,21]. The program is based on the Motivational Enhancement Therapy manual and Cognitive Behavioral Coping skills training manual used in project MATCH [18,19] and consists of 10 sessions of motivational interviewing, skills training and relapse prevention. In the first session, advantages and disadvantages of substance use are discussed. In the current study, the first session is also used to explain that substance use can cause symptoms that mimic ADHD symptoms. With motivational interviewing techniques, patients are motivated for abstinence in order to assess the effect of abstinence on their ADHD symptoms. As soon as a patient is motivated to become abstinent, or at least reduce substance use, procedures and self control measures on how to achieve this goal are discussed. Also risk factors for using substances (e.g. meeting certain persons, being in certain places or having certain feelings) are identified. These first four sessions (treatment phase 1) are the same for all participating patients, i.e. independent of the treatment condition after randomization. Subsequently, the diagnostic assessment of ADHD is repeated, and if the ADHD diagnosis is confirmed, randomization takes place. The remaining six sessions in the TAU only condition are used for a range of SUD treatment interventions. A functional analysis of the substance abusing behavior is made, strategies are trained to cope with craving, dealing with lapses and preventing relapse, and social refusal skills are offered. In the ninth session the patient can repeat one of the coping skills or choose one of several optional topics, depending on the specific needs of the patient. The treatment is concluded with an evaluation.

Participants in the experimental treatment condition receive an integrated treatment for SUD and ADHD, combining the main elements of the CBT program for SUD with CBT interventions for ADHD from the ‘Mastering your adult ADHD’ program developed by Safren et al. [22,23]. The original treatment program by Safren et al. [22] focuses on the training of coping skills and on symptom management strategies. It consists of 12 sessions, divided into three core modules, two optional modules, and a closing session. The first module (four sessions) focuses on psycho-education about ADHD and several organization and planning skills, such as using a calendar and task list system, problem solving by generating alternatives and picking the best solution, and breaking down complex or overwhelming tasks into smaller steps. The second module (two sessions) focuses on reducing distractibility by removing sources of distraction during a task, or by writing down distractions versus acting on them. The third module (three sessions) involves cognitive restructuring and adaptive thinking. Optional modules can be used to tackle procrastination and to involve a family member for support. The final session is used for evaluation and relapse prevention.

For the integrated treatment condition, the ADHD treatment program and the CBT program for the treatment of substance use disorders were integrated in the following way. We adapted the original ADHD treatment into a more condensed version, in which the core elements of planning and organization skills are presented to the patients in five sessions. In another six sessions from the CBT program directed at the treatment of SUD, 15 minutes are used each session to discuss homework assignments on the ADHD themes and to evaluate the learned skills. Similarly, in the five ADHD sessions, 15 minutes are used to discuss homework assignments on SUD themes. In this way, attention to the training of planning and organizational skills is given in 11 sessions in total. The contrast between the two approaches thus concerns five extra sessions ‘net-time’ on ADHD related issues in the integrated treatment protocol, plus the fact that ADHD symptoms are at least briefly discussed in 11 sessions. Sessions are planned weekly on a fixed time and day as much as possible. The outlines of the control condition (TAU only) and integrated treatment protocol are described in Table 1.

**Table 1:** Treatment programs

	**Treatment as Usual (TAU) only**	**Integrated treatment protocol**
Session 1	Introduction, advantages and disadvantages of substance use, effect of substance use on mental problems, enhancing motivation to become abstinent	Introduction, advantages and disadvantages of substance use, effect of substance use on mental problems, enhancing motivation to become abstinent
Session 2	Treatment goals and treatment plan	Treatment goals and treatment plan
Session 3	Self control measures	Self control measures
Session 4	Risk situations	Risk situations
Session 5	Analysis of functional elements in substance use	*ADHD: introduction of a cognitive model of ADHD, introduction of calendar and task list in notebook*
Session 6	Dealing with craving	Analysis of functional elements in substance use (similar to session 5 in TAU)
Session 7	Relapse and relapse prevention	*ADHD: problem solving*
Session 8	Social pressure	Dealing with craving (similar to session 6 in TAU)
Session 9	Optional theme: one of earlier themes can be repeated, or one of the themes ‘changing of thoughts’ or ‘dealing with emotions’ can be explored.	*ADHD: reducing distractibility*
Session 10	Evaluation	Relapse and relapse prevention (Similar to session 7 in TAU)
Session 11		*ADHD: mood problems*
Session 12		Social pressure (similar to session 8 in TAU)
Session 13		*ADHD: organizing papers*
Session 14		Optional theme: one of earlier themes can be repeated, or one of the themes ‘changing of thoughts’ or ‘dealing with emotions’ can be explored. (similar to session 9 in TAU)
Session 15		Evaluation (Similar to session 10 in TAU)

### Therapists

Both TAU only and integrated treatment sessions are delivered by therapists who received formal training and supervision in delivering protocolized CBT for SUD and have extensive experience of more than eight years. All therapists received four hours additional training for the CBT interventions targeting ADHD symptoms. Weekly supervision of both integrated treatments and TAU only by an experienced staff member (EV) is provided for the duration of the trial.

### Measurements

With the exception of some self-report questionnaires, most assessments are conducted in face-to-face contacts. The following instruments are used (see also Table 2):

**Table 2:** Measurement instruments

**Instrument**	**Screening and diagnostic assessment (t-1)**	**Baseline (t0)**	**Repetition of ADHD diagnostic assessment (t1)**	**Outcome (t2)**	**Follow up (t3)**
CIDI	•				
ASRS	•				
CAADID	•		•		
MSI-BPD	•				
SCID II Borderline	If MSI-BPD > 6				
ADHD rating scale		•		•	•
TLFB		•	•	•	•
BDI, BAI		•		•	•
EQ-5D		•		•	•
TIC-P		•		•	•
Computerized tests: BART, Stroop and Tower of London		•		•	•
Urine test and breath analysis		•		•	•

*Abbreviations*: *CIDI*, Composite International Diagnostic Interview; *ASRS*, Adult ADHD Self Report Scale; *CAADID*, Conners’ Adult ADHD Diagnostic Interview for DSM-IV; *MSI-BPD*, McLean Screening Instrument for Borderline Personality Disorder; *SCID II*, Structured Clinical Interview for DSM-IV Axis II; *TLFB*, Time Line Follow Back; *BDI*, Beck Depression. Inventory; *BAI*, Beck Anxiety Inventory; *EQ-5D*, a measure of health status from the EuroQol Group; TIC-P, questionnaire for costs associated with psychiatric illness (in Dutch); *BART*, Balloon Analogue Risk Task; Stroop, Stroop Color-Word task; *TOL*, Tower of London.

#### Selection and diagnostic measurements

1. The DSM-IV diagnosis of SUD is made using a Dutch questionnaire based on the Composite International Diagnostic Interview (CIDI vs 2.1) [26].

2. Screening for the presence of ADHD is performed with the first six items of the Adult ADHD Self-Report Scale (ASRS v1.1) [27,28].

3. The DSM-IV diagnosis of ADHD is based on a semi-structured interview, the Conners’ Adult ADHD Diagnostic Interview for DSM-IV (CAADID) [29].

4. Screening for the presence of a Borderline Personality Disorder is performed with the McLean Screening Instrument for Borderline Personality Disorder (MSI-BPD) [30].

5. In case of a score of seven or higher on the MSI-BPD, diagnostic evaluation of a Borderline Personality Disorder is performed with the corresponding module of the Structured Interview for DSM-IV Axis II (SCID II) [31,32].

The first two instruments were already implemented on the research site because they were used in an international study on the prevalence of ADHD in adults seeking treatment for substance use disorders (IASP study: International ADHD in Substance use disorder Prevalence study) [33], developed and executed by the International Collaboration on ADHD and Substance Abuse (ICASA), in which the treatment centre of the current study participated.

The ASRS has been validated for use in the general adult population [27] and some small validity studies are available for SUD patients [34-36]. Meanwhile, the IASP study has collected data on the validity of this instrument in a SUD population showing adequate sensitivity and specificity even in patients still actively using substances [37].

The CAADID consists of two parts. In the first part of the CAADID, information is obtained on the developmental history of the patient. This part can be filled out by the patient, preferably with the help of parents, before coming to the diagnostic appointment. In the second part of the CAADID, the presence of all ADHD symptoms is checked for both childhood and adulthood. Although it is generally recommended to include an interview with a family member in the diagnostic procedure in order to obtain information on childhood behaviors, we chose not to include such an interview in our diagnostic procedure. This choice was made because of logistic reasons and also because we encounter a lot of patients who have lost contact with their relatives due to their substance use disorder. Moreover, the study by Murphy and Schachar showed that adult patients can give a true account of both childhood and current symptoms of ADHD [38]. Another complexity of the diagnostic procedure in this patient population is the fact that we perform the diagnostic assessment of ADHD at a moment in time (during the intake phase) where most patients are still actively abusing substances. This may bias the results of the assessment, but for early detection and effective treatment allocation it is necessary to perform the diagnostic assessment in an early stage. In order to obtain a reliable and valid ADHD assessment, we chose to repeat the diagnostic interview for ADHD (CAADID) after four sessions of SUD treatment. We expect that most patients will have reached abstinence or achieved a substantial reduction of substance use by that time and that intoxication and withdrawal symptoms will no longer influence the assessment. If a diagnosis of ADHD is not confirmed at that moment, the patient is excluded from further participation in the second treatment phase of the study and receives regular TAU for SUD.

The MSI-BPD yielded both good sensitivity (0.81) and good specificity (0.85) in a non-substance abusing population when using a cut-off value of 7 [30]. In case of a positive screening result on the MSI-BPD, diagnostic evaluation of Borderline Personality Disorder is performed. Patients with a diagnosis of Borderline Personality Disorder are excluded from the study and allocated to adequate treatment.

#### Outcome measures

1. The ADHD rating scale is used as the measure for severity of ADHD symptoms [39], Dutch version [40,41]. In the Dutch version, the ADHD rating scale is a 23 item self-report questionnaire in which each item is scored on a 4-point scale ranging from 0 to 3.

2. The Time Line Follow Back (TLFB) [42-44] is used as a self-report measure for alcohol consumption and other substance use. In this study, it is used to assess alcohol and drug use in the past two months. From this questionnaire, several scores can be derived; in the statistical analyses we use a combined measure of percentage days of excessive use in the past two months for the primary drug of abuse, defined as at least six standard units of alcoholic beverages for men per day, at least four standard units of alcoholic beverages for women (in the case of alcohol as the primary drug of abuse) per day, more than one joint (in the case of cannabis as the primary drug of abuse) per day, or any use of other illicit drugs.

3. The Beck Depression Inventory (BDI) [45] is used as self-report questionnaire to measure the presence and severity of current depressive symptoms. It consists of 21 items in which each item is scored on a 4-point scale ranging from 0 to 3. Similarly, the Beck Anxiety Inventory (BAI) [46] is used to measure the presence and severity of anxiety symptoms. The BAI consists of 21 items scored on a 4-point scale ranging from 0 to 3.

4. The EQ-5D is a five-dimensional instrument for measuring quality of life [47,48]. The EQ-5D has shown to be valid in heroin dependent and in alcohol dependent patients [49,50]. It is used for cost-utility analyses.

5. The TIC-P is a questionnaire measuring the costs associated with psychiatric illness [51]. It has been used in many studies about patients with mental disorders (including schizophrenia, depression and ADHD) to estimate direct medical costs and costs due to production losses in order to perform cost-utility and cost-benefit analyses [52-56].

6. Computerized neuropsychological tests are performed to objectively assess (changes in) ADHD associated neuropsychological functions.

a. The Balloon Analogue Risk Task (BART) [57] provides information on risk taking behavior. This task can give information on subtypes of ADHD patients within a SUD population, differentiating between patients with high and low scores on risk taking behavior.

a. The Stroop Color-Word task [58-60] provides information on interference control. Patients with ADHD have repeatedly been reported to be impaired on this task [61,62]. Atomoxetine has been reported to improve the function on this task in adult patients with ADHD [63]. It is currently unknown if a planning and organization intervention can improve the performance on this task in ADHD patients.

a. The Tower of London task [64] is performed to measure planning ability. Patients with ADHD are reported to have impaired functions of planning. Methylphenidate can improve this function in adult ADHD patients [65] and it is interesting to learn if a planning and organization intervention can improve the performance on this task too.

7. Results of urine samples and breath analyses are used to provide additional, objective information on substance use.

#### Primary and secondary outcome measures

The primary outcome measure is the difference in the severity of ADHD symptoms according to the ADHD rating scale [40] between the integrated treatment condition and the TAU only condition at the end of the treatment (t1).

Key secondary outcome measures are the difference in the severity of ADHD symptoms between the two conditions at follow up two months after end of treatment (t2), and the difference of percentage of treatment responders (defined as a reduction of at least 30% of ADHD symptoms [15,16,66,67]) between TAU only and the integrated treatment condition at end of treatment and at follow up. The difference of TLFB scores between the two conditions at end of treatment and at follow up is another key secondary outcome measure.

Other secondary outcome measures are the differences in scores on the BDI, BAI, EQ-5D, TIC-P, Stroop task and Tower of London task at end of treatment and at follow up.

### Statistical analysis

Data will be analyzed according to the intention-to-treat principle. The effect of the integrated treatment in terms of the primary outcome variable will be assessed with generalized linear mixed model regression analysis (GLMM) with (Y_tj_-Y_t1_), j = 2,3 as dependent variable vector; treatment condition, time and the treatment condition by time interaction as predictor, and Y_t1_ (i.e. baseline value) as covariate. This model assumes missing at random (MAR), which adjusts the effect estimates for potential bias due to differential loss to follow-up. GLMM will also be used for the analysis of the secondary outcome variables, except the binary treatment responder outcome, for which a Generalized Estimating Equation model (GEE) will be used.

Costs will be calculated using standard costs for economic evaluations in health care [68]. The cost-effectiveness ratio is defined as the ratio of incremental mean costs and the incremental mean effect of the integrated treatment versus TAU only. Bootstrapping will be used to calculate the confidence interval for this ratio. In addition, a cost-utility analysis will be performed with data of the costs and quality of life of participants in both treatment conditions.

All analyses will be performed with SPSS 20 (SPSS Inc, Chicago, IL, USA).

#### Sample size

The primary outcome is the difference in the severity of ADHD symptoms according to the ADHD rating scale [40] between the experimental condition (integrated treatment) and the control condition (TAU only) at the end of the treatment (t2) using an ITT analysis and GLMM to account for missing values. In the study by Safren et al. [15] among ADHD patients without SUD, the between group standardized effect size (d) in terms of ADHD symptom scores was 0.60. We expect a somewhat smaller effect size because the population of patients with ADHD and SUD has potentially more complex problems, the intervention in the current study is a shorter version than the original one, and the contrast between the two conditions (integrated treatment and TAU) is smaller, because CBT is used in both conditions. On the other hand, time to follow up is shorter in the current study. Therefore, we estimate the effect size at d = 0.5 (medium effect). Based on a two-sided alpha = 0.05 and power = 0.80, 65 patients are needed in each condition. In order to adjust for loss of power due to an anticipated drop out of 15%, 150 participants will be included.

### Ethical review and trial registration

This RCT has been reviewed and approved by the ethics committee of the Academic Medical Centre in Amsterdam (number 10–130). It is registered in http://www.clinicaltrials.gov as NCT01431235.

## Discussion

To the best of our knowledge, this is the first randomized controlled trial to test the efficacy of an integrated CBT protocol aimed at reducing SUD as well as ADHD symptoms in a SUD population with comorbid adult ADHD. In the absence of effective pharmacological treatment options for ADHD in these patients, it is vital that other treatment options such as CBT are tested. Recent results of CBT interventions in ADHD patients, as shown by Safren et al. [15], Solanto et al. [16] and Emilsson et al. [17], are quite encouraging and we hope to find positive results in this dual disorder patient population as well by adding a CBT program directed at ADHD symptoms to an existing evidence-based CBT directed at SUD treatment.

In the regular practice of our SUD treatment centre, abstinence of substances is pursued first, and referral to an ADHD outpatient clinic is generally effectuated at a later stage. However, ADHD patients are often not identified in this population whereas ADHD symptoms often interfere with SUD treatment, for example in the most elemental way by forgetting appointments with therapists due to a lack of organization skills. This results in less optimal SUD treatment outcomes and very low numbers of patients actually receiving adequate ADHD treatment. We hypothesize that integrating treatment of ADHD symptoms with SUD treatment will show a better outcome. Two major challenges in both treatment and research of these patients with complex disorders are the tendency to relapse into substance use and the problems with planning and treatment adherence. In fact, we envisage that the greatest challenge of this study will be to prevent participants from dropping out of the study. We expect that SUD patients with comorbid ADHD will show higher levels of impulsivity and treatment drop-out, which requires a creative and persistent approach from both therapists and investigators. We addressed this by giving special attention to the format of the treatment: we chose for an individual version in order to allow for optimal flexibility of patient inflow and to avoid waiting lists. We also try to schedule the appointments in both treatment conditions at a fixed day and time to minimize the chance of forgetting appointments, and patients are reminded of their appointments by text messages sent the day before the appointment. Furthermore, in our power calculation we allowed for relatively high numbers of drop outs in phase 1 of the treatment. Patients who drop out of treatment in phase 2 of the treatment are still approached for outcome and follow up assessments; if patients are unwilling to come to the clinic for this purpose, a shortened version consisting of the ADHD rating scale and the Time Line Follow Back is administered by telephone in order to collect at least the most important data from as much participants as possible. We also expect that relapse into substance use, or inability to become abstinent, will be a common problem in both treatment conditions. Although treatments are aimed at full abstinence, relapse and continued substance use are no reason to end the treatment, but they will require therapists to use the treatment protocol in a flexible way. This can lead to diminished protocol adherence, but on the other hand enhances ecological validity of the study.

In this first trial, information is collected on acceptance, feasibility, efficacy and cost-effectiveness of the integrated treatment program compared to TAU only. It should be noted, however, that we adapted the original adult ADHD treatment program of Safren et al. [22,23] to a shorter version that could be closely integrated with the CBT treatment directed at reduction of SUD symptoms. In this shorter version of the Safren et al. protocol, the core skills of planning and organization, reducing distractibility, and coping with mood problems, are clearly present but they are offered in a tighter time frame and integrated with the existing treatment program for SUD that partly overlaps with some of these skills. However, several items from the original Safren et al. protocol are removed, for example the part on gauging your personal attention span. The part on cognitive restructuring is shortened, but in the SUD treatment sessions ample attention is given to this theme as well. In the integrated treatment condition, all sessions after randomization (sessions 5–15) are used to (also) evaluate ADHD skills. Overall, we believe that ADHD problems therefore receive ample attention in the integrated treatment. The result of our adaptations is a condensed version of the original CBT program for adult ADHD, still containing the core elements and covering the content of (a part of) 11 therapy sessions.

Patients who are already on a stable dose of ADHD medication at study entry, can participate in the study, but new medication is not provided to the participants. As efficacy of stimulants and other ADHD medication has not been shown in randomized trials yet, the efficacy of the current medication options is at least controversial. If patients want to start ADHD medication, they have several options: they can be included in the study if they agree to postpone medication use until the end of study (i.e. after the two months follow-up period) or they can refuse participation in this study and be referred to an ADHD treatment centre where medication can be prescribed. Of course, patients can withdraw from participation any time during the study and therapists can always refer a participant for a medication consult if they feel that medication is necessary.

An important difference between the current study and the study by Safren et al. [15] concerns the design of the control condition. As we want to know whether the integrated treatment has advantages over the treatment as usual, it is vital that we preserve the CBT program for SUD (TAU only) as the control condition in our design. This reduces the contrast between the two conditions, because in both conditions patients receive CBT. This is essentially different from a relaxation therapy used by Safren et al. [15] or support group (used by Solanto et al. [16]) as control condition, and reduces the probability of finding a positive result. At the same time, this makes the current design highly relevant for daily clinical practice as the difference between the two conditions in our study is made only by the number of sessions and the specific content of the CBT. Furthermore, the CBT program for SUD is the standard treatment of SUD in the Netherlands, and the merits of additional treatment components should be weighed against the results of this TAU program.

The unequal amount of therapy sessions that participants in the integrated treatment condition and in the TAU only condition receive is a limitation as well. Although we are aware that conclusions on the effectiveness of the CBT interventions for ADHD are limited because any effects, should they occur, can be attributed to having had more therapy time and attention in general, we decided in favor of this design as it is the first study on this issue in this patient population. Furthermore, the difference in time spent in therapy between the two conditions is relatively limited and may in reality even be smaller since not all patients will attend all sessions. In this first study we want to explore whether a combination of a little extra time with a more specific ADHD treatment is effective at all and whether this is cost-effective.

Another limitation of our design is the fact that assessors are not blinded for treatment allocation (and of course patients and therapists are not blinded either), because of logistic reasons. Again, we realize that this may introduce (information) bias. However, the presence of objective measures such as neuropsychological tests and biological markers of substance use will allow us to explore whether such bias really occurred and how such bias may have influenced the results.

Despite these challenges and limitations, we think that this study provides a first important step towards developing a tailored integrated treatment protocol for patients with SUD and comorbid ADHD.

## Competing interests

The authors declare that they have no competing interest.

## Authors’ contributions

KvE coordinates data-collection, was involved in preparing the integrated treatment protocol and drafted the research protocol and the current paper. EV was involved in preparing the integrated treatment protocol; supervises the clinical delivery of the treatments, supports the management of the project and critically revised the manuscript. WvdB contributed to the design of the study; supervises the management of the project and critically revised the manuscript. MWK provided statistical expertise to the protocol. KdB contributed to the preparation of the integrated treatment protocol. RAS contributed to the design and organization of the study and obtained funding; supervises the project and critically revised the manuscript. All authors have contributed to the writing of this paper and have read and approved the final paper.

## Pre-publication history

The pre-publication history for this paper can be accessed here:

http://www.biomedcentral.com/1471-244X/13/132/prepub
